# Dermatology Residency Selection Criteria with an Emphasis on Program Characteristics: A National Program Director Survey

**DOI:** 10.1155/2014/692760

**Published:** 2014-03-17

**Authors:** Farzam Gorouhi, Ali Alikhan, Arash Rezaei, Nasim Fazel

**Affiliations:** ^1^Department of Dermatology, University of California, Davis, 3301 C Street, Sacramento, CA 95816, USA; ^2^Department of Dermatology, Mayo Clinic, Rochester, MN, USA; ^3^Department of Civil & Environmental Engineering, University of California, Davis, USA

## Abstract

*Background*. Dermatology residency programs are relatively diverse in their resident selection process. The authors investigated the importance of 25 dermatology residency selection criteria focusing on differences in program directors' (PDs') perception based on specific program demographics. * Methods*. This cross-sectional nationwide observational survey utilized a 41-item questionnaire that was developed by literature search, brainstorming sessions, and online expert reviews. The data were analyzed utilizing the reliability test, two-step clustering, and *K*-means methods as well as other methods. The main purpose of this study was to investigate the differences in PDs' perception regarding the importance of the selection criteria based on program demographics. * Results*. Ninety-five out of 114 PDs (83.3%) responded to the survey. The top five criteria for dermatology residency selection were interview, letters of recommendation, United States Medical Licensing Examination Step I scores, medical school transcripts, and clinical rotations. The following criteria were preferentially ranked based on different program characteristics: “advanced degrees,” “interest in academics,” “reputation of undergraduate and medical school,” “prior unsuccessful attempts to match,” and “number of publications.”* Conclusions*. Our survey provides up-to-date factual data on dermatology PDs' perception in this regard. Dermatology residency programs may find the reported data useful in further optimizing their residency selection process.

## 1. Introduction

The dermatology residency application process is a highly competitive and daunting endeavor. An affirmation of this is the notable finding that applicants who have successfully matched into dermatology have the second highest average USMLE Step  1 scores amongst all residency applicants [1]. Dermatology applicants usually apply to an average number of 80 out of 114 available programs and approximately 25% of candidates will attend more than 21 interviews [1].

Dermatology programs are quite variable in their demography and characteristics and the leadership style as well as the philosophy behind the leadership can be extensively different in these programs. For example, few programs have officially added a significant dedicated time to research to their residency curriculum. As a recent example, The University of Texas Medical Branch has successfully incorporated research into resident's daily activity [2]. The literature in assessing different attributes of the dermatology programs as well as its correlation with residency selection process is lacking. Considering the competitive nature of the field and the diversity of residency programs in their selection criteria, gaining a better understanding of program priorities and selection criteria would be instrumental to the respective dermatology programs and prospective applicants. We hypothesized that the programs' characteristics may contribute to the PDs' perception about selection process. Therefore, we investigated the relative importance of 25 residency selection criteria among PDs of dermatology programs in the United States. This included an assessment of PDs' perception concerning completion of a fellowship (basic science or clinical) prior to residency training. Moreover, various correlations between the PDs' perception and the characteristics of their respective programs were investigated.

## 2. Methods

### 2.1. The Development and Utilization of the Questionnaire

This study was an online cross-sectional survey using an explicit questionnaire. A draft of the questionnaire was created by reviewing relevant published literature using PubMed and EMBASE [3, 4]. The initial draft was further developed by a brainstorming session and subsequently reviewed by five content experts, including three dermatology PDs, to generate the finalized questionnaire. The majority of the questions had a 1 to 10 analogue scale (10 = extremely important to 1 = not at all important, supplemental content 1, available online at http://dx.doi.org/10.1155/2014/692760). The survey was conducted via http://www.surveymonkey.com. The questionnaire (online appendix  1) contained a total of 41 items with 17 questions since one question included 25-item residency criteria.

The PDs from 114 accreditation council for graduate medical education ACGME-approved dermatology programs were included as eligible responders of this survey. The e-mail addresses of the PDs were obtained by a systematic search within the ACGME, American Medical Association (AMA), American Academy of Dermatology (AAD), and individual program websites. Residency coordinators and/or faculty members were contacted directly in instances where the systematic search was unsuccessful or the PD's e-mail address on record was no longer in use. A $5 Starbucks e-gift card accompanied the invitations. PDs who did not respond to the initial survey request were contacted via e-mail up to four additional times to improve compliance. Each PD had a unique uniform resource locator (URL) to access the survey.

The study protocol was approved by the IRB committee at the University of California, Davis with exemption.

### 2.2. Primary and Secondary Outcomes

The main purpose of this study was to investigate the differences in the importance of the selection criteria according to individual program characteristics. These characteristics consisted of a total number of research grants, number of editorial board members in faculty, total number of residents and faculties, faculty to resident ratios, and the availability of postgraduate fellowships and research track positions.

The secondary outcome was to assess the general PDs' perceptions regarding the relative importance of a 25-item set of residency selection criteria. We also explored the relative importance of the following factors in residency selection: source or content of letters of recommendation (LOR), the nature of a publication/presentation, and the field of research/publication.

### 2.3. Additional Retrieval of Program Attributes

Program attributes were extracted by searching the FRIEDA and ACGME database. They included the availability of postresidency fellowships such as pediatric dermatology, procedural dermatology, and dermatopathology.

### 2.4. Number of Residents and Faculty Members

The total number of filled residency positions and research track positions were retrieved by searching the ACGME website. Additionally, the total number of full-time faculty was determined by inquiry of the PDs. The total number of full-time faculty members on the editorial boards in the top 20 dermatology journals (according to the ISI Web of Knowledge Journal Citation Reports) was retrieved by reviewing the journals.

### 2.5. Research Grants

The total number and amount of National Institute of Health (NIH, 2007–2011) grants were accessed by searching the NIH RePORTER website. Dermatology foundation (DF, 2007–2011), National Rosacea Society (2006–2011), National Alopecia Areata Foundation (2006–2011), National Psoriasis Foundation (2007–2011), and Skin Cancer Foundation (2006–2011) grants were retrieved by reviewing the relevant websites or contacting the corresponding organization via e-mail.

### 2.6. Statistical Analysis

**Figure 1:**
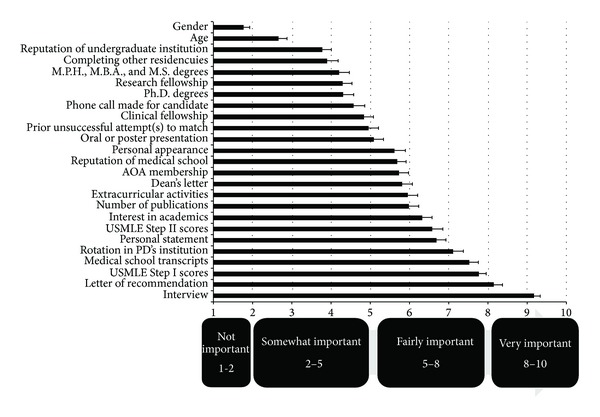
The graphical demonstration of the ranks of the 25-item residency selection criteria.

PASW statistics 18 (SPSS Inc., Chicago, IL, USA) was utilized. Statistical significance was generally defined as a *P* value ≤ 0.05. Continuous variables were presented as the means ± SEM. The normality of these variables was tested by the Kolmogorov-Smirnov test. Due to the abnormal distribution of the variables with 1–10 analogue scale, the Mann-Whitney test was applied for comparisons within different selection criteria based on program characteristics. For categorical variables, the *χ* ^2^ and Fisher exact tests were used when appropriate. The *K*-means method was utilized to recluster the 1–10 scoring system to a more qualitative scoring system. As shown in Figure 1, the new clusters of the 1–10 analogue scale were calculated as follows: 1-2 as “not important,” 2–5 as “somewhat important,” 5–8 as “fairly important,” and 8–10 as “very important.”

## 3. Results

Ninety-five out of 114 (83.3%) eligible dermatology PDs completed our survey. The internal consistency of the questionnaire was more than satisfactory with a Cronbach alpha of 0.861 when we assessed all 17 items of the questionnaire. Figure 1 demonstrates the graphical results of the residency selection criteria. Based on the present study, the top 5 residency selection criteria in order of importance include interview, letters of recommendation, USMLE Step I score, medical school transcripts, and rotation at the PD's institution.

When we asked about the source of an LOR, PDs considered a letter from someone they know closely (8.30 ± 0.19) of a greater importance than an LOR from a chair or PD (7.78 ± 0.24), a well-known dermatologist (7.04 ± 0.25), or a well-known expert in another field of medicine (5.58 ± 0.26). All comparisons reached statistical significance.

Peer reviewed publications (7.04 ± 0.26) were significantly preferred over oral presentations (5.97 ± 0.24), poster presentations (5.72 ± 0.23), and abstracts (5.64 ± 0.25). Although an oral presentation is likely to be more competitive, it was similarly weighted to a poster presentation in importance. When PDs were asked about the order of the authors in a publication, 44% considered a first author publication more favorably than a second-to-last author publication while 40% of PDs said that it depends on the quality of the paper.

**Figure 2:**
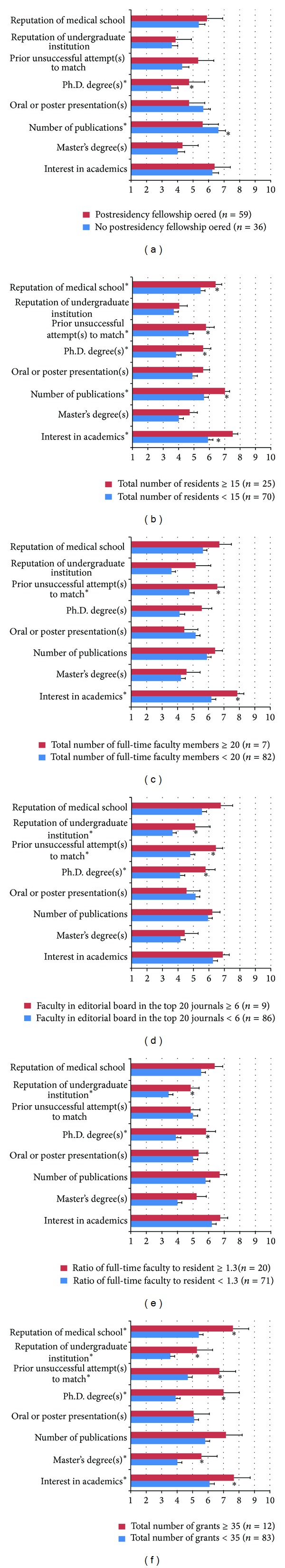
The relative importance of major academic criteria in dermatology residency selection after dichotomizing the results based on the programs that offered postresidency fellowships or not (a), the number of residents (b), the number of full-time faculty members (c), the number of faculty members on the editorial board in the top 20 dermatology journals (d), the ratio of (full-time) faculty/resident (e), and total number of grants (f). *The comparisons with asterisks are statistically significantly different from each other.

The two-step clustering method was utilized to compare program characteristics with the results of the residency selection criteria. The calculated cut points were 35 for the total number of grants, 7 for the number of non-NIH grants, 15 for the number of ACGME approved residency slots, 1.3 for the ratio of faculty to residents members, 20 for the number of full-time faculty members, 10 for the number of faculty members on a selection committee, and 6 for the number of editorial board members in the top 20 dermatology journals. The dichotomization data are presented in Figure 2.

**Figure 3:**
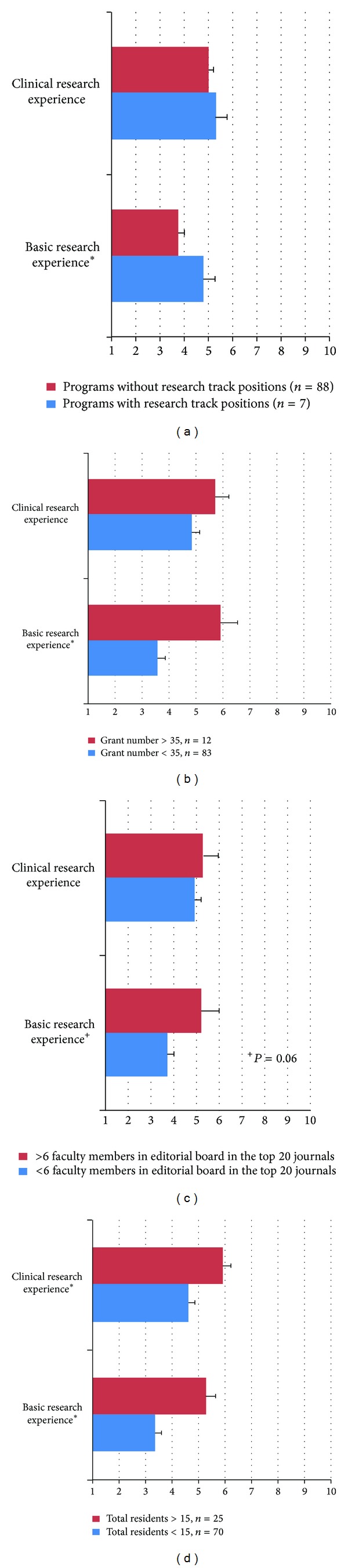
The differences in PD's attitude regarding the importance of basic and clinical research fellowship prior to beginning residency are shown. The results have been illustrated based on programs offering a research track position (a), number of grants (b), number of faculty members on the editorial board in top dermatology journals (c), and number of residents (d). *The comparisons with an asterisks reached to a statistical significance.

Specifically, completion of a clinical research fellowship was deemed more favorably than a basic science research fellowship (4.97 ± 0.23 versus 3.89 ± 0.26, resp., *P* < 0.05). However, programs with a larger number of residents and faculty members, those with a larger number of grants, and those that offered a research track position considered a basic science research fellowship comparable to a clinical fellowship (Figure 3).

Our data support two findings regarding specific areas of research. Firstly, dermatology research experience is preferable to research in other fields of medicine (5.13 ± 0.27 versus 3.93 ± 0.24, for single or few topics in dermatology versus other fields, *P* < 0.05). Secondly, having a focus on one or a few research topics was preferred over research on a wide range of topics (5.13 ± 0.27 versus 4.09 ± 0.27, for single or few topics versus wide range of topics in dermatology, *P* < 0.05). When we specifically asked about the importance of dermatology versus nondermatology research in the context of research fellowship training, the average importance was 4.71 ± 0.25, which fell into the category of “somewhat important.” The reputation of the institution in which the applicant participated in research was also considered as “somewhat important” with a mean score of 4.28 ± 0.24. Surprisingly, applicant research funding was not perceived as important (2.23 ± 0.20).

When survey responders expressed their preference about the length of research fellowship training, 47% did not have a preference while 43% of PDs favored a duration of 1 year or less.

## 4. Discussion

To our knowledge, this is the first study to investigate the differences between dermatology PDs' perceptions of the relative importance of residency selection criteria in detail based on distinctive program characteristics. The other strength of our survey is that it achieved a response rate of 83.3%. This is substantially higher than the 2012 NRMP PD survey [5], in which the response rate for dermatology PDs was 45.3%. According to our study, “interview” and “letters of recommendation” were the only factors ranked as “very important.” Many other factors were also deemed important. The 2012 NRMP dermatology PD survey results show some overlap with our study. This survey indicated that factors related to interview, interpersonal skills, evidence of professionalism and ethics, Dean's letter, grades in required clerkships, and letters of recommendation were all important factors in the selection of residency candidates [5]. Plastic surgery and orthopedic surgery, much like dermatology, are amongst the most competitive and highly sought after residencies in the US. Several of the same selection criteria overlap between dermatology, orthopedic surgery [4], and plastic surgery [3] PDs, including USMLE Step 1 score, grades, letters of recommendation, and rotation at the PD's program.

In our survey, interviews were the most important factor in residency candidate selection even though other factors may have been implemented before offering an interview to an applicant. This is self-explanatory because in order to receive an interview, one's application must be viewed favorably by the program and the applicant's merits are felt to be strong; hence, personal factors become more important. In general surgery programs, interview is perceived as the most important factor by PDs, chairs, and associate PDs [6]. A similar study of PDs of prosthodontic programs in dentistry also found that interviews were the most important factor when selecting candidates [7]. Furthermore, they found that the most important characteristics of the applicant considered during the interview were honesty, organization, energy, confidence, decision-making, and verbal skills. The same trend was observed in ophthalmic plastic and reconstructive surgery fellowship [8], ophthalmology [9], and otolaryngology [10]. Additionally, PDs of emergency medicine programs identified interviews as the second most important criterion [11]. Interviews, as with any other interpersonal interaction, can be biased and even potentially discriminatory as evidenced by the fact that almost all the dermatology residency applicants of one medical school were asked at least one discriminatory question during residency interviews [12]. Such biases can be minimized by using different methods like multiple mini-interviews (MMI) [13] although this method may have its own disadvantages [14, 15]. For example, pharmacy residents disagreed with the fact that MMI is more efficacious or less stressful than a traditional interview [16]. In a study, MMI method was compared to the traditional interview in a pool of interns applying to emergency medicine. Although MMI was perceived less favorably than traditional interview, MMI did correlate with emergency medicine clerkship grades as a residency selection criterion [17].

LORs were the second most important factor in residency candidate selection. These letters may have the ability to distinguish between competitive and noncompetitive applicants [18]. In terms of the source of the letter, PDs preferred letters written by dermatologists they know closely, followed by chairpersons and other PDs. Similar to our study, LORs from division chiefs were considered the most important followed by letters from clinical faculty amongst fellowship directors within the field of [16] pediatric emergency medicine [4]. Miller et al. suggested the relatively high importance of LORs written by a chair or a PD for dermatology residency applicants as compared to LORs by others [19]. Some specialties like emergency medicine [20–23] and otolaryngology [24, 25] have incorporated a standardized format for the LORs submitted by the applicants in an attempt to better assess the strengths and weaknesses of the prospective residents. This can be potentially used in dermatology.

In our study, similar to previous surveys, USMLE Step  1 scores were considered important. There has been much debate as to whether USMLE scores truly correlate with subjective clinical skill acquisition or residency performance [26–30], though there may be a moderate correlation with performance on dermatology in-training exams [31]. Interestingly, in a recent meta-analysis involving a total of 41704 participants and 80 studies, USMLE scores were among the strongest predictors of current doctor's performance [32]. USMLE Step  1 scores are often the only standardized and universally available measure of academic performance and therefore a useful screening tool within a large pool of competitive candidates. Many programs utilize USMLE Step  1 score cutoffs to initially screen applicants and cut down on the large volume of applications that come in each year for a limited number of residency positions. This is of significant importance given that many candidates are encouraged to apply to a large number of programs because of the highly competitive nature of dermatology [1].

In our study, peer reviewed publications were perceived more favorably than meeting presentations while Poirier and Pruitt noted a comparable importance placed by the PDs for publication and presentation in pediatric emergency medicine fellowship [4].

A study of plastic surgery PDs found that the most important “subjective criterion” was the candidate's performance on away/subinternship rotation [33]. Interestingly, compared to dermatology PDs, emergency medicine PDs felt that USMLE scores were less important, while rotation grades in the specialty were deemed more important [11]. Rotation at the PD's institution was ranked as the fifth most important criterion in our study, thus emphasizing the importance of dermatology rotations [34]. Away rotations may be of greater significance in the dermatology application review process as compared to other larger specialties considering that dermatology programs have a limited number of residency slots and therefore any personality conflicts may have a larger impact on the overall cohesiveness of a relatively smaller cohort of residents. The opportunity for greater interaction with the faculty and residents during the course of an away rotation can provide meaningful insight into whether or not a candidate will fit in well with the cohort of residents. Therefore, having the opportunity to get to know an applicant over the course of an away rotation rather than just the limited interaction during the course of a residency interview can be invaluable in the selection process.

Although not highly ranked on the list, “prior unsuccessful attempt to match” was perceived as a more important factor (presumably a negative factor) than an M.P.H., M.B.A., or M.S. degree, completing other residency training, and the reputation of the undergraduate institution. Stratman and Ness reported that the factors strongly associated with subsequent matching of applicants with an unsuccessful attempt included USMLE Step  3 score; LORs by academic dermatologists; completion of preliminary internships rather than transitional internship; publication record; and completion of non-ACGME approved dermatology fellowships [35].

Interesting differences were observed when program characteristics were taken into account. Advanced non-MD degrees, interest in academics, number of publications, completion of a research fellowship, reputation of undergraduate and medical school, and prior unsuccessful attempts to match were differentially ranked (Figures 2 and 3). It can be argued that the reputation of any given medical school is dependent on the degree of research conducted at the institution as is suggested by the annual* US News and World Report* rankings [36]. This finding suggests that applicants with advanced non-M.D. degrees (particularly Ph.D.) and those having completed a research fellowship would likely have a competitive edge over conventional M.D. applicants at least in some programs with more research focus. This may be partly explained by the more extensive record of scholarly achievement typically seen amongst Ph.D. candidates. A survey study of general surgery PDs demonstrated that 89.5% of respondents considered basic or clinical research “almost always” or “all the time” in the evaluation of their applicants [37].

Our data indicated that larger programs, those with more faculty members on editorial boards, those with more grant funding, and programs with postgraduate fellowships and research track positions gave greater importance to research experience and an interest in academic dermatology. These programs may have a stronger emphasis on research and more academic career opportunities.

It is possible that candidates express an interest in academics at the time of interview in order to improve their chances of matching. This is further supported by a study showing that indeed the positive predictive value of such professed interest is very low (8%) [38]. Although ranked as the eighth important factor in dermatology residency selection, it does not seem to be a reliable criterion since a prospective applicant's interests and priorities can change during the course of residency training [38, 39].

It is prudent to mention that if less important residency selection criteria indicate highly positive or negative attributes regarding any particular candidate, the effects of those relatively less important criteria may outweigh the impact of the top five. For example, a student with a* Nature* or* Science* publication will more than likely be considered a competitive candidate for a research track position or a student who has concerning comments in the Dean's letter may be less desirable independent of their credentials within the top five criteria.

A limitation to our study is that our questionnaire did not investigate the differences in the residency selection criteria for the initial screening of applicants versus subsequent selection processes. Additionally, the nature of a survey study may impose some limitations. Specifically, the results represent opinions. This makes the outcomes rather subjective.

## 5. Conclusions

Our survey provides up-to-date data on dermatology PDs' perceptions based on program characteristics and demographics. Thus, this will be useful to dermatology or other competitive similar residencies' PDs and their selection committees in comparing and potentially adjusting their selection preferences based on the aforementioned facts given the competitive nature of the specialty and the variability in program philosophies, resources, and needs.

## Supplementary Material

Supplemental Appendix 1:
This is the complete version of the questionnaire. We made it available to all the responders. It was conducted in an online format using http://www.surveymonkey.com/. The responders were asked 41 items in a 17-question format since one question included 25-item residency criteria.
