# Supplementary material for: Dermatology Residency Selection Criteria with an Emphasis on Program Characteristics: A National Program Director Survey
Source: Dermatol Res Pract. 2014 Mar 17;2014:692760. doi: 10.1155/2014/692760 (PMC3977115; doi:10.1155/2014/692760)
Supplement: Supplementary file 1 — Supplemental Appendix 1: This is the complete version of the questionnaire. We made it available to all the responders. It was conducted in an online format using http://www.surveymonkey.com/. The responders were asked 41 items in a 17-question format since one question included 25-item residency criteria. [file 692760.f1.docx]

**Program Directors Survey**

[**Exit this survey**](http://www.surveymonkey.com/s.aspx?PREVIEW_MODE=DO_NOT_USE_THIS_LINK_FOR_COLLECTION&sm=re5pDgpn9uvQUjlQX1DY1oGIGIwXQVkKNt6i8CW%2bQao%3d)

**1. How many faculty members are actively involved with your residency program? 
Current number of faculty members:**

| Fulltime: |  |
| --- | --- |
| Part time: |  |
| Volunteer: |  |
| Total: |  |

**2. How many of your faculty members and/or staff participate in the selection of residents for your program?**

**3. Please identify your program if you want?**

**4. Which of the following are generally most important factors for you to decide to select a dermatology applicant? For each of the items listed below, please select a number on a scale of 1 to 10 scale as: (1=Not at all important to 10=Extremely important) Then of all the items listed below, please indicate the three most important items to you at the end of the question.**

|  | 1 | 2 | 3 | 4 | 5 | 6 | 7 | 8 | 9 | 10 |
| --- | --- | --- | --- | --- | --- | --- | --- | --- | --- | --- |
| a. Age |  |  |  |  |  |  |  |  |  |  |
| b. Alpha Omega Alpha (AOA) membership |  |  |  |  |  |  |  |  |  |  |
| c. Clinical Fellowships |  |  |  |  |  |  |  |  |  |  |
| d. Completing Other Residency Program (e.g. internal medicine, pathology, pediatrics) |  |  |  |  |  |  |  |  |  |  |
| e. Dean’s Letter |  |  |  |  |  |  |  |  |  |  |
| f. Extracurricular Activities (Volunteer and community service) |  |  |  |  |  |  |  |  |  |  |
| g. Gender |  |  |  |  |  |  |  |  |  |  |
| h. Interest in Academics |  |  |  |  |  |  |  |  |  |  |
| i. Interview |  |  |  |  |  |  |  |  |  |  |
| j. Letters of Recommendation |  |  |  |  |  |  |  |  |  |  |
| k. Medical School Transcripts |  |  |  |  |  |  |  |  |  |  |
| l. MPH, MBA or MS degrees |  |  |  |  |  |  |  |  |  |  |
| m. Number of Publications |  |  |  |  |  |  |  |  |  |  |
| n. Oral or Poster Presentation |  |  |  |  |  |  |  |  |  |  |
| o. Personal Appearance |  |  |  |  |  |  |  |  |  |  |
| p. Personal Statements |  |  |  |  |  |  |  |  |  |  |
| q. PhD Degrees |  |  |  |  |  |  |  |  |  |  |
| r. Prior Unsuccessful Attempt(s) to match into Dermatology residency |  |  |  |  |  |  |  |  |  |  |
| s. Reputation of Applicant’s Undergraduate Institution |  |  |  |  |  |  |  |  |  |  |
| t. Reputation of Applicant’s Medical School |  |  |  |  |  |  |  |  |  |  |
| u. Research Fellowships (pre-residency) |  |  |  |  |  |  |  |  |  |  |
| v. Rotation in your Institution |  |  |  |  |  |  |  |  |  |  |
| w. USMLE Step I Scores |  |  |  |  |  |  |  |  |  |  |
| x. USMLE Step II Scores |  |  |  |  |  |  |  |  |  |  |
| y. Telephone Call Made on Behalf of the Candidate |  |  |  |  |  |  |  |  |  |  |

Please specify your top three items in order (e.g. c, f, i)

**5. How important is basic science research experience in the selection of residency applicants? (1=Not at all important- 10=Extremely important)**

| 1 | 2 | 3 | 4 | 5 | 6 | 7 | 8 | 9 | 10 |
| --- | --- | --- | --- | --- | --- | --- | --- | --- | --- |

**6. How important is clinical science research experience in the selection of residency applicants? (1=Not at all important-10=Extremely important)**

| 1 | 2 | 3 | 4 | 5 | 6 | 7 | 8 | 9 | 10 |
| --- | --- | --- | --- | --- | --- | --- | --- | --- | --- |

**7. How important is it that an applicant’s research be dermatology related rather than non-dermatology related research when considering his/her residency application? (1=Not at all important-10=Extremely important)**

| 1 | 2 | 3 | 4 | 5 | 6 | 7 | 8 | 9 | 10 |
| --- | --- | --- | --- | --- | --- | --- | --- | --- | --- |

**8. How important is applicant research funding in your decision to select? (1=Not at all important-10=Extremely important)**

| 1 | 2 | 3 | 4 | 5 | 6 | 7 | 8 | 9 | 10 |
| --- | --- | --- | --- | --- | --- | --- | --- | --- | --- |

**9. How important is the reputation of the institution in which the applicant did research? (1=Not at all important-10=Extremely important)**

| 1 | 2 | 3 | 4 | 5 | 6 | 7 | 8 | 9 | 10 |
| --- | --- | --- | --- | --- | --- | --- | --- | --- | --- |

**10. For applicants with research experience, how important are the following in your selection of residency applicants? (1-10 scale for each choice, 1. Not at all important to 10. Extremely important)**

|  | 1 Not at all important | 2 | 3 | 4 | 5 | 6 | 7 | 8 | 9 | 10 Extremely important |
| --- | --- | --- | --- | --- | --- | --- | --- | --- | --- | --- |
| Applicant’s research has been published as a paper in a peer reviewed journal |  |  |  |  |  |  |  |  |  |  |
| Applicant has published a research meeting abstract |  |  |  |  |  |  |  |  |  |  |
| Applicant has presented his or her research as an oral presentation at a national or regional scientific meeting |  |  |  |  |  |  |  |  |  |  |
| Applicant has presented his or her research as a poster at a national or regional scientific meeting |  |  |  |  |  |  |  |  |  |  |

Other (please specify) and rank from 1 to 10

**11. How important are the following in your selection of residency applicants with multiple research publications? (1-10 scale for each choice, 1. Not at all important to 10. Extremely important)**

|  | 1. Not at all important | 2 | 3 | 4 | 5 | 6 | 7 | 8 | 9 | 10 Extremely important |
| --- | --- | --- | --- | --- | --- | --- | --- | --- | --- | --- |
| Publications on a wide range of topics within the field of medicine |  |  |  |  |  |  |  |  |  |  |
| Publications on a wide range of topics within the field of dermatology |  |  |  |  |  |  |  |  |  |  |
| Publications on a single topic or few related topics in medicine |  |  |  |  |  |  |  |  |  |  |
| Publications on a single topic or few related topics in dermatology |  |  |  |  |  |  |  |  |  |  |
| Do not consider publications for residency application |  |  |  |  |  |  |  |  |  |  |

Other (please specify) and rank from 1 to 10

**12. Which applicant do you prefer? The applicant with…:**

| 2nd author to last author publication(s) | First author publication(s) | Depends on the quality | Not a factor |
| --- | --- | --- | --- |

**13. What type of recommendation letter will have greatest impact on your decision to select a residency applicant with research experience? (1-10 scale for each choice, 1. No impact to 10. Extremely large impact)**

|  | 1 Not at all important | 2 | 3 | 4 | 5 | 6 | 7 | 8 | 9 | 10 Extremely important |
| --- | --- | --- | --- | --- | --- | --- | --- | --- | --- | --- |
| Letters from dermatology program director or chairperson |  |  |  |  |  |  |  |  |  |  |
| Letters from a well-known dermatologist |  |  |  |  |  |  |  |  |  |  |
| Letters from a well-known expert in other fields of medicine |  |  |  |  |  |  |  |  |  |  |
| Letters from a person you know closely |  |  |  |  |  |  |  |  |  |  |
| Letters written very strongly |  |  |  |  |  |  |  |  |  |  |
| Letters that are completely fair and honest |  |  |  |  |  |  |  |  |  |  |

**14. Which, in your opinion, is the ideal length of research experience prior to starting dermatology residency training?**

| 1 month | 2-11 months | 1 year | 2 years | >2 years | No preference |
| --- | --- | --- | --- | --- | --- |

**15. Do you prefer an applicant who has done research at multiple institutions over an applicant with research experience at one institution?**

| Yes | No | No preference |
| --- | --- | --- |
| Depends (Please describe): | | |

**16. Please name all residency selection committee members that are willing to participate in this survey. Thanks.**

**17. Would you like to have summarized results of this survey send to you?**

| Yes | No |
| --- | --- |

Thank you very much for your contribution. We really appreciate your commitment to the advancement of dermatology residency programs.
